# Protective Action of Antioxidants on Hepatic Damage Induced by Griseofulvin

**DOI:** 10.1155/2014/982358

**Published:** 2014-01-12

**Authors:** M. del C. Martinez, S. G. Afonso, A. M. Buzaleh, A. Batlle

**Affiliations:** ^1^Departamento de Química Biológica, Facultad de Ciencias Exactas y Naturales, Universidad de Buenos Aires, Intendente Guiraldes 2160 Pab II, 1428 Buenos Aires, Argentina; ^2^Centro de Investigaciones Sobre Porfirinas y Porfirias (CIPYP), CONICET, Hospital de Clínicas José de San Martín, Universidad de Buenos Aires, Avenida, Córdoba 2351 1er subsuelo, 1120 Buenos Aires, Argentina

## Abstract

Erythropoietic protoporphyria (EPP) is a disease associated with ferrochelatase deficiency and characterized by the accumulation of protoporphyrin IX (PROTO IX) in erythrocytes, liver, and skin. In some cases, a severe hepatic failure and cholestasis were observed. Griseofulvin (Gris) develops an experimental EPP with hepatic manifestations in mice such as PROTO IX accumulation followed by cellular damage as wells as necrotic and inflammatory processes. The antioxidant defense system was also altered. The aim was to evaluate the possible protective effect of different antioxidant compounds: trolox (Tx), ascorbic acid (Asc), the combination Tx and Asc, melatonin (Mel), and the polyphenols: ellagic acid, quercetin, chlorogenic acid, caffeic acid, gallic acid, and ferulic acid on liver damage and oxidative stress markers in a mouse model of EPP. Coadministration of Gris with Tx, Asc, and its combination, or Mel mainly affected heme biosynthetic pathway, resulting in a decrease in ALA-S activity which was increased by Gris, while the tested polyphenols exerted a protective effect on oxidative stress, decreasing lipid peroxidation and the activity of some antioxidant enzymes. In conclusion, antioxidant compounds can only protect partially against the liver damage induced by Gris, reducing oxidative stress or acting on heme regulation.

## 1. Introduction 

Erythropoietic protoporphyria (EPP) is a disease associated with a diminished activity of ferrochelatase (FECH) (EC.4.99.1.1), the final enzyme of heme biosynthesis that catalyzes the conversion of protoporphyrin (PROTO IX) into heme [1, 2]. As a result, PROTO IX accumulates in bone marrow, erythrocytes, liver, and skin [3, 4]. The most serious manifestation of this porphyria is the progressive liver failure, cholestasis, and deposition of PROTO IX in the canalicular bile. There is a correlation between the importance of the liver damage and PROTO IX levels in erythrocytes and in some cases the injury is so severe that it could require liver transplantation [5, 6].

The antifungal griseofulvin (7-chloro-4,6-methylspiro trimetoxi-6-[benzophenone-2 (3H),1-(2) cyclohexene] 3, 4-dione; Gris) develops a model of EPP with hepatic manifestations in animals [7–10]. Previously, we have demonstrated that the administration of Gris to mice produced PROTO IX accumulation followed by cellular damage and necrotic and inflammatory processes [11]. These alterations were similar to those found in the human EPP associated with liver failure. Furthermore, the development of oxidative stress was observed, so liver redox balance was altered due to porphyrin high concentrations, known generators of reactive oxygen species (ROS). As a consequence, the antioxidant defense system was disrupted and reflected by an increased activity of the enzymes glutathione reductase (GRed), superoxide dismutase (SOD) and glutathione-S-transferase (GST), high levels of reduced glutathione (GSH), and malondialdehyde (MDA), as well as a reduced activity of glutathione peroxidase (GPx) and catalase [11].

Several studies have been reported in both patients and animal models concerning the use of antioxidants like vitamins A, E, and C or melatonin (Mel) on acute and cutaneous porphyrias [12–17]. Vitamin E (*α*-tocopherol) is the main antioxidant compound found in membranes, which controls the formation of lipid hydroperoxides through its antioxidant function [18, 19]. Trolox (Tx) is a hydrosoluble analogue of *α*-tocopherol; the difference in their solubility is due to the replacement of a phytol chain by a carboxyl group [20]. It has been shown that this compound, like vitamin E, has antioxidant action against lipid peroxidation and its use as a possible therapeutic agent for the prevention of tissue damage mediated by free radicals in relevant clinical situations has been suggested [21, 22].

Ascorbic acid (Asc) is considered a powerful antioxidant, scavenging ROS and reactive nitrogen species. Although it is a hydrophilic molecule, it is involved in the protection of hydrophobic compounds such as membrane lipids, through a cooperative action with vitamin E. Asc acts regenerating alpha-tocopherol from alpha-tocopheryl radical [23, 24]. So, the antioxidant power of vitamin E is increased by the presence of vitamin C.

Mel is a well-known antioxidant, free radical scavenger, and a powerful protective agent under several experimental conditions [25, 26]. It can stimulate the activity of some antioxidant enzymes such as SOD, GRed, and GPx [26].

Polyphenols are compounds widely distributed in the plant kingdom, generally involved in the defense against UV radiation or aggression by pathogens [27]. The most abundant are phenolic acids, flavonoids, stilbenes, and lignans; of these, flavonoids and phenolic acids account for 60% and 30%, respectively, of total polyphenols in the diet, approximately 1 g/day [28]. The derivatives of the most abundant phenolic acids in plants are hydroxybenzoic and hydroxycinnamic acids. Hydrobenzoic acid content in edible plants is generally very low, except for certain fruits and vegetables such as radishes and onions. A representative of this group is gallic acid, and its most important source is tea. Hydroxycinnamic acids are more common than hydrobenzoic acids and they are very abundant in most fruits and coffee. To this group belong caffeic acid (CfA), ferulic acid, (FA) and chlorogenic acids (CA) (formed by combination of caffeic and quinic acids). Flavonols are the most ubiquitous flavonoids in foods; one of the more known is quercetin (Que). The richest sources of Que are onion, leek, broccoli, and blueberries. Red wine and tea also contain large amounts of this polyphenol. Ellagic acid (EA) is another flavonoid mainly present in nuts and fruits like grapes, strawberries, raspberries, blackberries, and blueberries.

In vitro and in vivo studies showed that these compounds have antioxidant properties including scavenging of ROS and lipid peroxidation inhibition, exhibiting also anticancerinogenic antimutagenic, antibacterial, antiviral, and anti-inflammatory effects [29].

Considering the damage produced by Gris in mice liver, the aim of these studies was to evaluate the ability of some antioxidant agents, namely, Tx, Asc, the combination, Tx and Asc, Mel of and the polyphenols: EA, Que, CA, CfA, GA, and FA, either preventing the Gris induced oxidative stress or at least improving the oxidative balance in the liver of Gris treated animals.

## 2. Materials and Methods 

### 2.1. Animals

Male mice CF1 weighing 15–17 g at the time of starting intoxication were used. Animals were maintained in controlled conditions and allowed free access to food (Purina 3, Asociación de Cooperativas Argentinas, San Nicolás, Buenos Aires, Argentina) and water. A 12/12 h light/dark cycle was maintained. Experiments were performed at the same time of the day. Animals received human care and were treated in accordance with the guidelines established by the Committee of the Argentine Association of Specialists in Laboratory Animals (AADEALC).

### 2.2. Experimental Design

In each experiment, animals were separated into 4 groups of 6 mice each. All animals received control diet (standard diet supplemented with corn oil, 10 mL/100 g). Gris was added to food and the antioxidants studied were added to water or they were injected via ip as indicated in the following protocol:

*group I:* control diet (standard diet supplemented with corn oil, 10 mL/100 g),

*group II:* control diet plus Gris (0.5% w/w),

*group III:* control diet plus Tx (2 mg/100 mL), Asc (12 mg/100 mL), Tx (2 mg/100 mL) plus Asc (12 mg/100 mL), EA (300 mg/L), Que (50 mg/L), CA (50 mg/L), CfA (650 mg/L), GA (50 mg/L), FA (60 mg/L) or Mel (5 mg/kg ip, 72, 48, 24, or 1 hour before to sacrifice),

*group IV:* control diet with Gris (0.5% w/w) plus Tx (2 mg/100 mL), Asc (12 mg/100 mL), Tx (2 mg/100 mL) plus Asc (12 mg/100 mL), EA (300 mg/L), Que (50 mg/L), CA (50 mg/L), CfA (650 mg/L), GA (50 mg/L), FA (60 mg/L), or Mel (5 mg/kg i.p, 72, 48, 24, or 1 hour before to sacrifice).

After 2 weeks of treatment, food was removed 16 h before the sacrifice of the animals under ether anesthesia. The liver was immediately processed.

### 2.3. Tissue Preparation

A fraction of the liver was cut with scissors and immediately homogenized (1 : 3, w/v) in a solution containing 0.9% NaCl, 0.1 mM Tris HCl, pH 7.4, and 0.5 mM EDTA, for ALA-S activity determination. The remainder tissue, previously perfused with ice cold saline, was removed. A fraction was homogenized (1 : 3, w/v) in ice cold 0.25 M sucrose. After differential centrifugation of the homogenate (10,000 ×g, for 20 min.), the supernatant was used for measuring GSH levels and GST activity. An aliquot of this supernatant was then centrifuged at 105,000 ×g, for 60 min and in this supernatant SOD activity was measured. Another fraction of the perfused liver was homogenized (1 : 10 w/v) in 0.05 M sodium phosphate buffer, pH 7.4, and it was used directly for the determination of MDA level or it was centrifuged for 10 min at 10,000 ×g. The resulting supernatant was used for measuring GRed activity. Another fraction of the perfused liver (200–300 mg) was homogenized in 2 mL of a homogenizing medium consisting of 8 parts of methanol: DMSO (4 : 1 v/v), and 1 part of water; it was centrifuged at 2,600 ×g for 10 min and the soluble fraction was used for measuring PROTO IX levels.

### 2.4. Biochemical Assays

PROTO IX levels were determined fluorometrically (*λ*ex 400 nm, *λ*em 632 nm) according to Polo et al. [30] with slight modifications.

*δ*-Aminolevulic acid synthetase (ALA-S) activity was measured following the method of Marver et al. [31]. Lipid peroxidation was estimated as MDA levels using the method of Ohkawa et al. [32]. GSH was quantified according to Rossi et al. [33]. GST activity was determined by the method of Habig et al. [34]. GRed was calculated using the method of Pinto and Bartley [35] and SOD by the method of Paoletti et al. [36]. Protein concentration was determined by the procedure of Lowry et al. [37].

Enzyme units were defined as the amount of enzyme that catalyses the formation of 1 nmol of product under the standard incubation conditions. One unit of SOD is defined as the amount of SOD causing 50% inhibition on the rate of NADH oxidation measured in the control. Specific activity was expressed as units/mg protein.

### 2.5. Statistical Analysis

All data represent mean values ± standard deviation (SD) of four experiments performed in duplicate. The differences between treated and control groups were determined by analysis of the variance (ANOVA) and the significance level was verified by the Bartlet test. A probability level of 99.9% or 99.5% was considered as significant difference between groups.

## 3. Results

### 3.1. Effect of Antioxidants on Heme Metabolism

**Figure 1:**
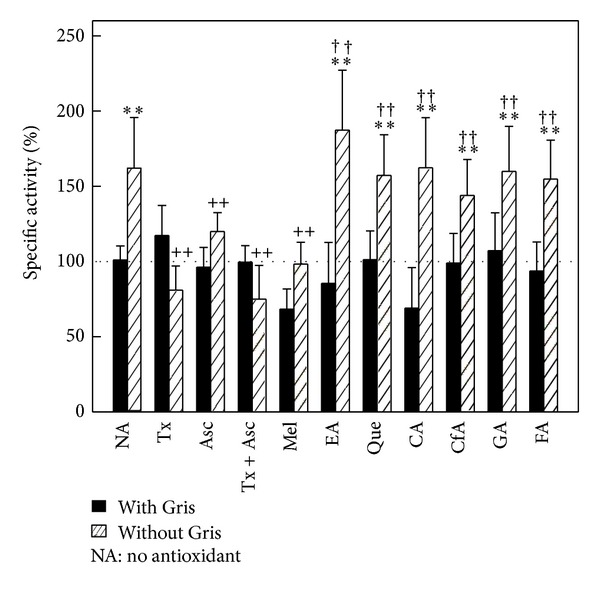
Effect of antioxidants on ALA-S activity. Mice received standard diet containing Gris (0.5%, w/w) and antioxidants in drinking water: Tx (2 mg/100 mL), Asc (12 mg/100 mL), Tx (2 mg/100 mL) plus Asc (12 mg/100 mL), EA (300 mg/L), Que (50 mg/L), CA (50 mg/L), CfA (650 mg/L), GA (50 mg/L), FA (60 mg/L), or Mel (5 mg/kg ip, 72, 48, 24, or 1 hour before sacrifice); over 14 days. Control group received only standard diet in oil (vehicle used for solubilised Gris). Results are expressed as percentage ± SD of 6 mice of the corresponding control value taken as 100% (dotted line). Mean control value: 0.144 ± 0.021 nmol/mg protein. **P < 0.01: significant difference between the group treated with Gris alone or Gris plus antioxidant and the control group. ^++^ *P* < 0.01: significant difference between the group treated with Gris plus antioxidant and the group that only received Gris. ^††^ *P* < 0.01: Significant difference between the group treated with Gris plus antioxidant and the group only treated with antioxidant. Experimental details are described in Section 2.

ALA-S activity was 60% (*P* < 0.01) increased in animals receiving Gris. The administration of Asc plus Gris decreased 25% (*P* < 0.01) this induction, while Tx, Tx plus Asc, or Mel returned the activity to basal levels (Figure 1). No significant changes were observed in the groups receiving the polyphenols.

None of the antioxidants studied produced significant differences in the content of PROTO IX in the liver (Table 1).

### 3.2. Effect of Antioxidants on Liver Damage Markers

**Figure 2:**
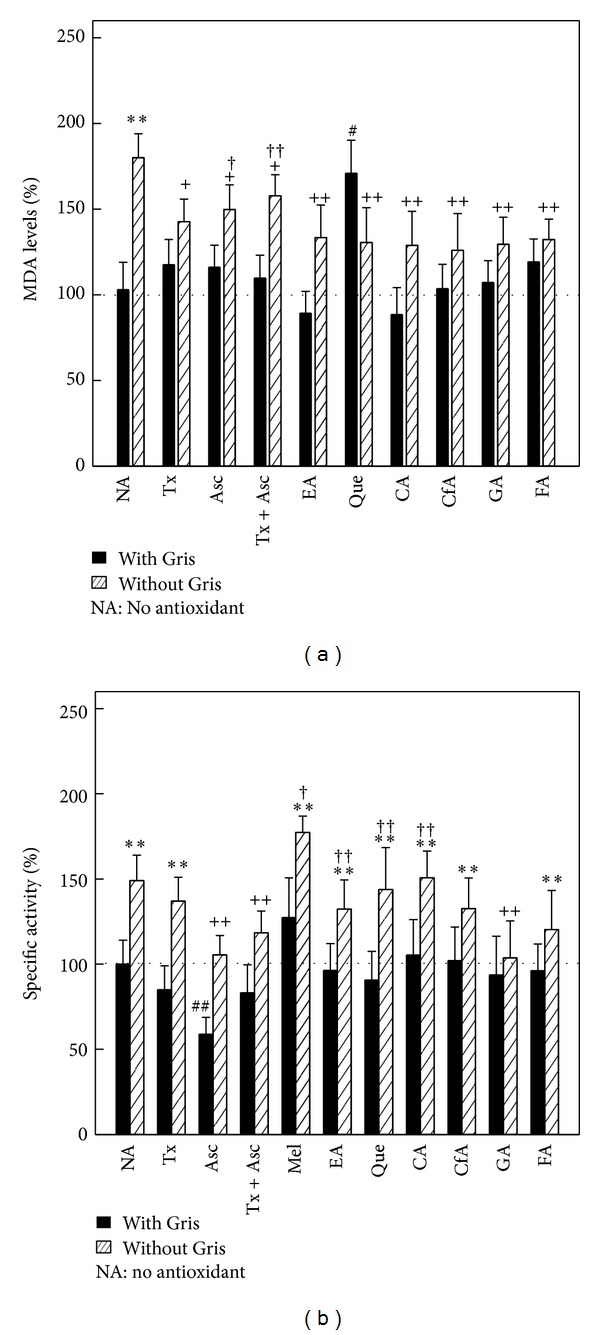
Effect of antioxidants on markers of liver damage. (a) MDA levels, mean control value: 3.15 ± 0.40 nmol/mg protein (n-6). (b) GSH levels, mean control value: 22.98 ± 5.29 nmol/mg protein (n-6). **P < 0.01: significant difference between the group treated with Gris alone or Gris plus antioxidant and the control group. ^+^ *P* < 0.05 and  ^++^ *P* < 0.01: significant difference between the group treated with Gris plus antioxidant and the group that only received Gris. ^#^ *P* < 0.05 and ^##^ *P* < 0.01: significant difference between the group treated with antioxidants and the control group. ^†^ *P* < 0.05 and ^††^ *P* < 0.01: significant difference between the group treated with Gris plus antioxidant and the group only treated with antioxidant. Experimental details are described in legend to Figure 1 and in Section 2.

Que was the only antioxidant increasing 70% (*P* < 0.01) the levels of MDA in control animals. Gris increased 80% (*P* < 0.01) lipid peroxidation with respect to control group. The combined treatment of Gris with the nonpolyphenolic antioxidants produced a decrease of around 18–20% (*P* < 0.05) in lipid peroxidation compared with the group receiving only Gris. All polyphenols administered in conjunction with Gris decreased 30% (*P* < 0.01) MDA levels, compared with the increase induced by Gris alone, but never reaching baseline levels (Figure 2(a)).

Asc administered to control animals was the antioxidant that reduced 40% (*P* < 0.01) GST activity. Gris induced 50% (*P* < 0.01) GST activity, keeping its activity elevated also after Tx, Mel, and all polyphenols with the exception of GA. Instead, when Asc or Asc plus Tx or GA was given to Gris fed mice, GST activity returned to control values (Figure 2(b)).

### 3.3. Effect of Antioxidants on Antioxidant Defense System

**Figure 3:**
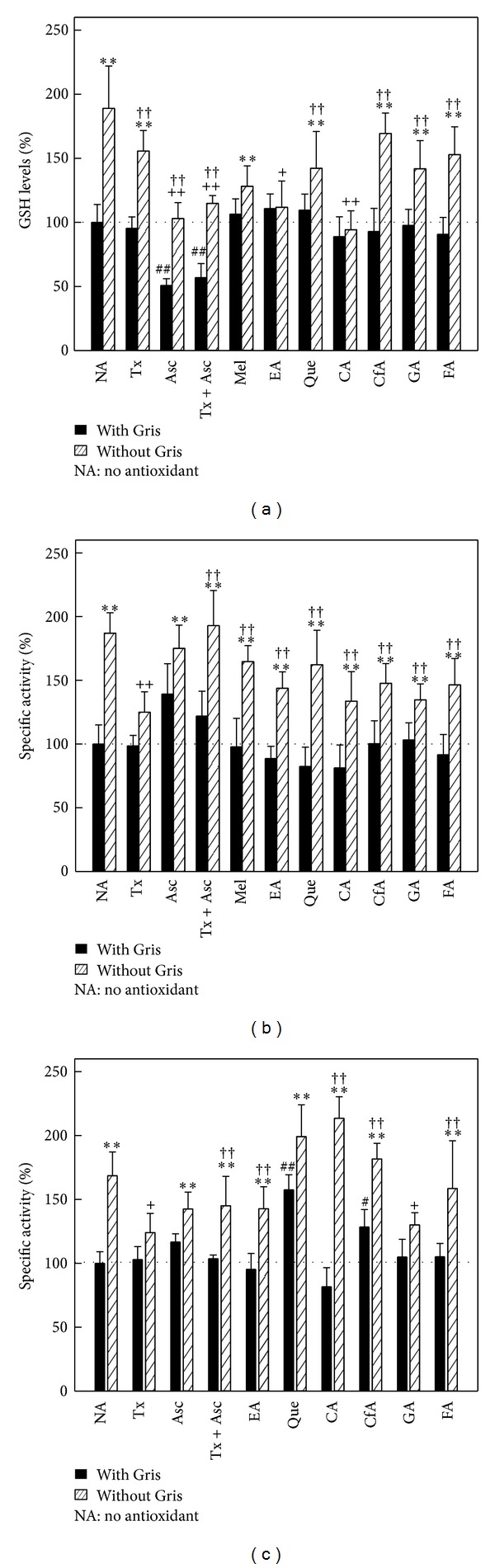
Effect of antioxidants on liver antioxidant defense system. (a) GRed, mean control value: 43.52 ± 7.40 nmol/mg protein (n-6); (b) SOD, mean control value: 83.90 ± 8.66 nmol/mg protein (n-6); and (c) GST, mean control value: 22.77 ± 1.20 *μ*nmol/mg protein (n-6). **P < 0.01: Significant difference between the group treated with Gris alone or Gris plus antioxidant and the control group. ^+^ *P* < 0.05 and ^++^ *P* < 0.01: significant difference between the group treated with Gris plus antioxidant and the group that only received Gris. ^#^ *P* < 0.05 and ^##^ *P* < 0.01: significant difference between the group treated with antioxidants andthe control group. ^†^ *P* < 0.05 and ^††^ *P* <0.01: significant difference between the group treated with Gris plus antioxidant and group only treated with antioxidant. Experimental details are described in legend to Figure 1 and in Section 2.

GSH content decreased by 55% (*P* < 0.01) when Asc alone or in combination with Tx was administrated to control animals, while all the other studied compounds had no effect at all on GSH levels. Gris treatment increased 90% (*P* < 0.01) hepatic GSH content, which was not altered by the administration of Tx, Mel, Que, CfA, GA, or FA. In animals receiving Gris with Asc, Asc plus Tx, CA, or EA, a reduction above 50% (*P* < 0.01) was detected, finally abolishing the effect of Gris (Figure 3(a)).

There were no significant changes in GRed activity when control animals were given antioxidants. The 87% (*P* < 0.01) induction of this enzyme activity due to Gris was reduced to 33% (*P* < 0.01) by Tx, without any effect when the other antioxidants were assayed (Figure 3(b)).

SOD activity increased 57% (*P* < 0.01) and 28% (*P* < 0.05) in the control group by the effect of Que and CfA, respectively, while no changes were detected after administration of the other antioxidants tested. Gris induced 75% (*P* < 0.01) SOD activity, but combining Gris with Tx o GA decreased by 30% (*P* < 0.01) induction of Gris alone. On the other hand, in animals treated with CA and Gris SOD activity increased 17% (*P* < 0.05) compared to the Gris fed group (Figure 3(c)).

## 4. Discussion

Gris is metabolized by cytochrome P450 producing N-methyl porphyrins, inhibitors of FECH. There was an accumulation of hepatic porphyrins and the concomitant induction of ALA-S activity in animals receiving this drug [1, 38, 39]. High levels of free radicals and lipid peroxidation indicate the induction of oxidative stress produced by this porphyrinogenic agent. The increase in ROS would be responsible for alterations in the antioxidant defense system, while increased GSH levels and SOD activity make up for the oxidative damage.

To investigate the possibility of avoiding the effect of Gris on heme metabolism and on the development of oxidative stress, we evaluated the action of different antioxidant compounds in liver mice fed with Gris.

The coadministration of Gris with Tx, Asc, Tx plus Asc or Mel mainly affected heme biosynthetic pathway, resulting in a decrease in ALA-S activity, 60% increased by Gris alone; on the other hand, the polyphenols assayed exerted a protective action on oxidative stress by decreasing lipid peroxidation levels and the activity of some antioxidant enzymes.

It has been demonstrated that Tx can protect the cells against the damage induced by oxidants [40]. The mechanism of Tx protection involves the kidnapping of oxygen-derived free radicals generated in the cell membrane lipids, very likely in a similar way to the action of *α*-tocopherol [41], in spite of the differences in the solubility between the two compounds. Gerez et al. [42] showed that *α*-tocopherol is able to reverse the induction of ALA-S and GST produced by hepatocarcinogenic agents.

Although several publications reported that Mel is a potent scavenger of free radicals and that it has antioxidant properties [26, 43, 44], in this study, we have not found any substantial changes in the activity of the antioxidant enzymes that could indicate that Mel was able to restore the antioxidant defense mechanism of the cell in Gris fed mice or to have any protective action against the Gris induced damage.

Different studies confirmed the potent activity of the polyphenols' compounds assayed on lipid peroxidation and the abduction of radical H_2_O_2_ [45–47]; our results were in agreement with those reports.

Hepatic GSH levels, increased by the effect of Gris, returned to control values when the animals received both Gris and EA or Que. We have previously shown that the activity of GRed was greatly increased in animals treated with Gris [11]. We have also found that the free pool of heme and catalase and GPx activity were greatly diminished, attributing the reduction of these parameters to a reduced synthesis of hemeproteins. This would also explain the increase in GSH levels observed in these animals. Therefore, the effect of these acids on GSH levels would not be due to an increase in the consumption of the thiol by GPx, because they did not affect the activity of catalase and GPx reduced by Gris (data not shown); therefore, preventing lipid peroxidation would occur by a different mechanism of action.

SOD activity significantly increased in all animals receiving Gris or Gris with polyphenols. Although the polyphenols may be free radical scavengers, these compounds were not acting on superoxide radicals.

In conclusion, treatment with antioxidants partially prevents liver damage induced by Gris, either decreasing oxidative stress, increasing excretion of porphyrins, or regulating heme metabolism.

Because oxidative stress observed in animals treated with Gris is mainly induced by porphyrins accumulated in liver and the polyphenols which protected the liver only against lipid peroxidation, further studies, to assess whether the simultaneous administration of the polyphenol and the drug could accelerate porphyrin excretion, are needed.

## Conflict of Interests

M. del C. Martinez is a researcher at the University of Buenos Aires. S. G. Afonso was a principal research assistant at the Argentine National Research Council (CONICET). A. M. Buzaleh and A. Batlle hold the post of Independent and Superior Scientific Researchers at CONICET.

## Figures and Tables

**Table 1:** Effect of antioxidants on liver PROTO IX levels.

Gris	PROTO IX levels (ng/mg protein)										
	Control	Tx	Asc	TX + Asc	Mel	EA	Que	CA	CfA	GA	FA
Without	0.42 ± 0.04	0.45 ± 0.06	0.41 ± 0.05	0.43 ± 0.04	0.44 ± 0.03	0.45 ± 0.01	0.41 ± 0.03	0.44 ± 0.03	0.47 ± 0.05	0.36 ± 0.04	0.48 ± 0.03
With	20.32 ± 3.15**	19.85 ±2.28^∗∗††^	21.01 ± 3.51^∗∗††^	20.40 ± 3.24^∗∗††^	17.88 ± 3.78^∗∗††^	22.63 ± 4.21^∗∗††^	18.62 ± 3.84^∗∗††^	19.76 ± 4.02^∗∗††^	24.16 ± 5.20^∗∗††^	19.57 ± 3.65^∗∗††^	21.96 ± 3.98^∗∗††^

Mice received standard diet containing Gris (0.5%, w/w); in the drinking water, they also received Tx (2 mg/100 mL), Asc (12 mg/100 mL), Tx (2 mg/100 mL) plus Asc (12 mg/100 mL), ellagic acid (EA, 300 mg/L), Que (50 mg/L), CA (50 mg/L), CfA (650 mg/L), GA (50 mg/L), FA (60 mg/L), or Mel (5 mg/kg ip, 72, 48, 24 or 1 hour before sacrifice), over 14 days. Control group received only standard diet of oil (vehicle used for solubilised Gris). Data represent mean values ± SD of 6 mice. **P < 0.01: significant difference between the group treated with Gris alone or Gris plus antioxidant and the control group. ^††^ *P* < 0.01: significant difference between the group treated with Gris plus antioxidant and the group only treated with antioxidant. Other experimental details are described in Section 2.

## References

[1] Batlle A (1997). *Porfirinas Y Porfirias. Aspectos Clínicos*.

[2] Lecha M, Puy H, Deybach J-C (2009). Erythropoietic protoporphyria. *Orphanet Journal of Rare Diseases*.

[3] Thunell S, Harper P, Brun A (2000). Porphyrins, porphyrin metabolism and porphyrias. IV. Pathophysiology of erythyropoietic protoporphyria—Diagnosis, care and monitoring of the patient. *Scandinavian Journal of Clinical and Laboratory Investigation*.

[4] Cox TM, Kadish KM, Smith KM, Guilard R (2003). Protoporphyria. *The Porphyrin Handbook. Medical Aspects of Porphyrias*.

[5] Meerman L (2000). Erythropoietic protoporphyria: an overview with emphasis on the liver. *Scandinavian Journal of Gastroenterology*.

[6] Bruguera M, Herrero C (2005). Liver disease in erythropoietic protoporphyria. *Gastroenterologia y Hepatologia*.

[7] Shapiro SH, Wessely Z (1984). Ultrastructural changes of intrahepatic bile ductules in griseofulvin fed mice. *Annals of Clinical and Laboratory Science*.

[8] Tanaka K, Ohgami T, Nonaka S (1993). Experimental murine protoporphyria induced by griseofulvin (GF): the relationship between hepatic porphyrin levels and liver function test values in mice treated with GF. *Journal of Dermatology*.

[9] Polo CF, Buzaleh AM, Vazquez ES, Afonso SG, Navone NM, Del Carmen Batlle AM (1997). Griseofulvin-induced hepatopathy due to abnormalities in heme pathways. *General Pharmacology*.

[10] Inafuku K, Takamiyagi A, Oshiro M, Kinjo T, Nakashima Y, Nonaka S (1999). Alteration of mRNA levels of *δ*-aminolevulinic acid synthase, ferrochelatase and heme oxygenase-1 in griseofulvin induced protoporphyria mice. *Journal of Dermatological Science*.

[11] Martinez MDC, Afonso SG, Meiss RP, Buzaleh AM, Batlle A (2009). Hepatic damage and oxidative stress induced by griseofulvin in mice. *Cellular and Molecular Biology*.

[12] Thunell S, Andersson D, Harper P, Henrichson A, Floderus Y, UlfLindh U (1997). Effects of administration of antioxidants in acute intermittent porphyria. *European Journal of Clinical Chemistry and Clinical Biochemistry*.

[13] Princ FG, Maxit AG, Cardalda C, Batlle A, Juknat AA (1998). In vivo protection by melatonin against *δ*-aminolevulinic acid-induced oxidative damage and its antioxidant effect on the activity of haem enzymes. *Journal of Pineal Research*.

[14] Alemzadeh R, Feehan T (2004). Variable effects of beta-carotene therapy in a child with erythropoietic protoporphyria. *European Journal of Pediatrics*.

[15] Anderson KE (2007). Porphyria cutanea tarda: a possible role for ascorbic acid. *Hepatology*.

[16] Székely E, Vereckei A, Almási A (2007). Effects of vitamin E administration on the hemorheological status and redox homeostasis of patients with porphyria cutanea tarda treated with phlebotomy. *Clinical Hemorheology and Microcirculation*.

[17] Ferrer MD, Tauler P, Sureda A, Palacín C, Tur JA, Pons A (2010). Variegate porphyria induces plasma and neutrophil oxidative stress: effects of dietary supplementation with vitamins e and C. *British Journal of Nutrition*.

[18] Brigelius-Flohé RB-F, Traber MG (1999). Vitamin E: function and metabolism. *FASEB Journal*.

[19] Cuzzocrea S, Thiemermann C, Salvemini D (2004). Potential therapeutic effect of antioxidant therapy in shock and inflammation. *Current Medicinal Chemistry*.

[20] Poljšak B, Raspor P (2008). The antioxidant and pro-oxidant activity of vitamin C and trolox in vitro: a comparative study. *Journal of Applied Toxicology*.

[21] Wu T-W, Hashimoto N, Au J-X, Wu J, Mickle DAG, Carey D (1991). Trolox protects rat hepatocytes against oxyradical damage and the ischemic rat liver from reperfusion injury. *Hepatology*.

[22] Kam Ming Ko KMK, Pak Kin Yick PKY, Poon MKT, Siu Po Ip SPI (1994). Prooxidant and antioxidant effects of trolox on ferric ion-induced oxidation of erythrocyte membrane lipids. *Molecular and Cellular Biochemistry*.

[23] Carr A, Frei B (1999). Does vitamin C act as a pro-oxidant under physiological conditions?. *FASEB Journal*.

[24] Kojo S (2004). Vitamin C: basic metabolism and its function as an index of oxidative stress. *Current Medicinal Chemistry*.

[25] Ng TB, Liu F, Zhao L (2000). Antioxidative and free radical scavenging activities of pineal indoles. *Journal of Neural Transmission*.

[26] Reiter RJ, Tan D-X, Osuna C, Gitto E (2000). Actions of melatonin in the reduction of oxidative stress: a review. *Journal of Biomedical Science*.

[27] Manach C, Scalbert A, Morand C, Rémésy C, Jiménez L (2004). Polyphenols: food sources and bioavailability. *The American Journal of Clinical Nutrition*.

[28] Scalbert A, Johnson IT, Saltmarsh M (2005). Polyphenols: antioxidants and beyond. *The American Journal of Clinical Nutrition*.

[29] Nichenametla SN, Taruscio TG, Barney DL, Exon JH (2006). A review of the effects and mechanisms of polyphenolics in cancer. *Critical Reviews in Food Science and Nutrition*.

[30] Polo CF, Frisardi AL, Resnik ER, Schoua AEM, del Batlle CAM (1988). Factors influencing fluorescence spectra of free porphyrins. *Clinical Chemistry*.

[31] Marver HS, Tschudy DP, Perlroth MG, Collins A (1966). Delta-aminolevulinic acid synthetase. I. Studies in liver homogenates. *The Journal of Biological Chemistry*.

[32] Ohkawa H, Ohishi N, Yagi K (1979). Assay for lipid peroxides in animal tissues by thiobarbituric acid reaction. *Analytical Biochemistry*.

[33] Rossi R, Cardaioli E, Scaloni A, Amiconi G, Di Simplicio P (1995). Thiol groups in proteins as endogenous reductants to determine glutathione-protein mixed disulphides in biological systems. *Biochimica et Biophysica Acta*.

[34] Habig WH, Pabst MJ, Jakoby WB (1974). Glutathione S transferases. The first enzymatic step in mercapturic acid formation. *The Journal of Biological Chemistry*.

[35] Pinto RE, Bartley W (1969). The effect of age and sex on glutathione reductase and glutathione peroxidase activities and on aerobic glutathione oxidation in rat liver homogenates. *Biochemical Journal*.

[36] Paoletti F, Aldinucci D, Mocali A, Caparrini A (1986). A sensitive spectrophotometric method for the determination of superoxide dismutase activity in tissue extracts. *Analytical Biochemistry*.

[37] Lowry OH, Rosebrough NJ, Farr AL, Randall RJ (1951). Protein measurement with the Folin phenol reagent. *The Journal of Biological Chemistry*.

[38] Frater Y, Brady A, Lock EA, de Matteis F (1993). Formation of N-methyl protoporphyrin in chemically-induced protoporphyria. *Archives of Toxicology*.

[39] Bellingham RMA, Gibbs AH, de Matteis F, Lian L-Y, Roberts GCK (1995). Determination of the structure of an N-substituted protoporphyrin isolated from the livers of griseofulvin-fed mice. *Biochemical Journal*.

[40] Wu T-W, Hashimoto N, Wu J (1990). The cytoprotective effect of Trolox demonstrated with three types of human cells. *Biochemistry and Cell Biology*.

[41] Burton GW, Ingold KU, Thompson KE (1981). An improved procedure for the isolation of ghost membranes from human red blood cells. *Lipids*.

[42] Gerez E, Vazquez E, Caballero F, Polo C, Batlle A (1997). Altered heme pathway regulation and drug metabolizing enzyme system in a mouse model of hepatocarcinogenesis: effect of veronal. *General Pharmacology*.

[43] Hardeland R, Pandi-Perumal SR (2005). Melatonin, a potent agent in antioxidative defense: actions as a natural food constituent, gastrointestinal factor, drug and prodrug. *Nutrition and Metabolism*.

[44] Tan D-X, Manchester LC, Terron MP, Flores LJ, Reiter RJ (2007). One molecule, many derivatives: a never-ending interaction of melatonin with reactive oxygen and nitrogen species?. *Journal of Pineal Research*.

[45] Tseng T-H, Wang C-J, Kao E-S, Chu H-Y (1996). Hibiscus protocatechuic acid protects against oxidative damage induced by tert-butylhydroperoxide in rat primary hepatocytes. *Chemico-Biological Interactions*.

[46] Raneva V, Shimasaki H, Ishida Y, Ueta N, Niki E (2001). Antioxidative activity of 3,4-dihydroxyphenylacetic acid and caffeic acid in rat plasma. *Lipids*.

[47] Sroka Z, Cisowski W (2003). Hydrogen peroxide scavenging, antioxidant and anti-radical activity of some phenolic acids. *Food and Chemical Toxicology*.

